# Management of stretched scar– induced secondary strabismus

**DOI:** 10.1186/s12886-020-01339-7

**Published:** 2020-02-19

**Authors:** Mohamed F. Farid, Mohamed R. Mahmoud, Mohamed A. Awwad

**Affiliations:** 1 Department of ophthalmology, Benha University, Benha, Egypt; 2 Department of ophthalmology, Ain Shams University, Ain Shams, Egypt; 3 Flat 7 1,30 Widmore Road, Bromley, BR1 3BE UK

**Keywords:** Stretched scar, Secondary strabismus, Consecutive strabismus, Recurrent strabismus

## Abstract

**Background:**

To determine characteristics and management of consecutive or recurrent strabismus secondary to stretched scar.

**Methods:**

This is a retrospective review of all patients diagnosed with late secondary consecutive or recurrent strabismus due to stretched scar from 2012 to 2017. The diagnosis of stretched scar was made in any case of late (≥ 1 month) consecutive or recurrent strabismus associated with underaction of the previously operated muscle. The diagnosis was confirmed intraoperatively by negative forced duction test and the characteristic appearance of the scar tissue. Surgical correction involved excision of the scar tissue with muscle re-attachment to the sclera using non-absorbable sutures. Study parameters include improvement in secondary deviations, degree of muscle underaction and diplopia.

**Results:**

21 consecutive and 6 recurrent cases of stretched scar –induced strabismus were identified and all cases were associated with variable degrees of limited ocular duction. After surgical correction of the stretched scar, consecutive deviations in the form of consecutive esotropia and exotropia were corrected by means of 26.1PD and 65.6PD while recurrent deviations in the form recurrent exotropia and recurrent hypertropia were corrected by means of 34.3PD and 11PD respectively with significant improvement of limited ocular ductions. 21 patients had diplopia at presentation and all were improved after surgery.

**Conclusion:**

management of stretched scar –induced secondary strabismus by excision of the stretched scar and muscle fixation to the sclera using non-absorbable sutures significantly corrects secondary deviations and improves limitation of ocular duction.

## Background

Secondary strabismus in the form of consecutive or residual strabismus is a well-known complication of strabismus surgery [1]. It has been reported that rates of undercorrection following surgery for esotropia and exotropia varied from 20 to 40% and from 22 to 59% respectively [2–5] whereas rates of overcorrection after surgery for esotropia and exotropia varied from 20 to 27% and from 2 to 20% respectively [6–10]. As secondary strabismus - either recurrent or consecutive- may occur long after good initial results, true rates of secondary misalignment are often underestimated [11, 12].

Secondary strabismus which occurs in the early postoperative period is proposed to result from muscle slippage and is commonly associated with severe underaction of the involved muscle [13]. Late secondary strabismus associated with variable degrees of muscle underaction which occurs months up to years from the primary procedure is thought to result from improper wound healing with stretching of the scar tissue intervening between normal muscle tissue and the sclera. The stretched scar causes weakening of the muscle action with development of secondary strabismus associated with weak duction in the direction of action of the involved muscle. This phenomenon was first explained by Ludwig and Chow in1999 through intraoperative observations and histological examinations and they called it the “stretched scar syndrome” [11, 12].

In ophthalmic literature, there are few clinical trials concerning the issue of stretched scar as a cause of secondary strabismus. The aim of this study is to present our experience with stretched scar induced – secondary strabismus regarding its clinical characteristics and outcomes of management.

## Methods

This is a retrospective review of patients who underwent reoperation from 2012 to 2017 for correction of secondary strabismus due to stretched scar by the means of exploration, excision of scar tissue and scleral attachment of the involved muscle(s) using non-absorbable sutures. Cases of secondary strabismus which needed additional surgeries of ipsilateral and/or contralateral muscles were also included.

The data collection conformed to the local laws and followed the tenets of the Declaration of Helsinki. Data collection included historical information regarding the primary procedure when applicable such as patient’s age at the primary surgery, side and type of the primary operation, time elapsed between the primary procedure and appearance or symptoms of secondary deviation. All patients were subjected to complete ophthalmic and orthoptic examinations which included best corrected visual acuity (BCVA), cycloplegic refraction and fundus examination. Assessment of angle of deviation was performed by prism and alternate cover test (PACT) both at distant (6 m) and near (1/3 m) fixation. Assessment of limited duction was performed and any limitation was recorded on a 6-point scale with − 6 was given to eyes with no movement, − 5 to eyes which moved towards the midline but were unable to reach it, − 4 to eyes which reached the midline but were unable to proceed further [14]. Using the slit lamp, carful inspection of the conjunctiva and the insertion area of extraocular muscles was performed.

**Fig. 1:**
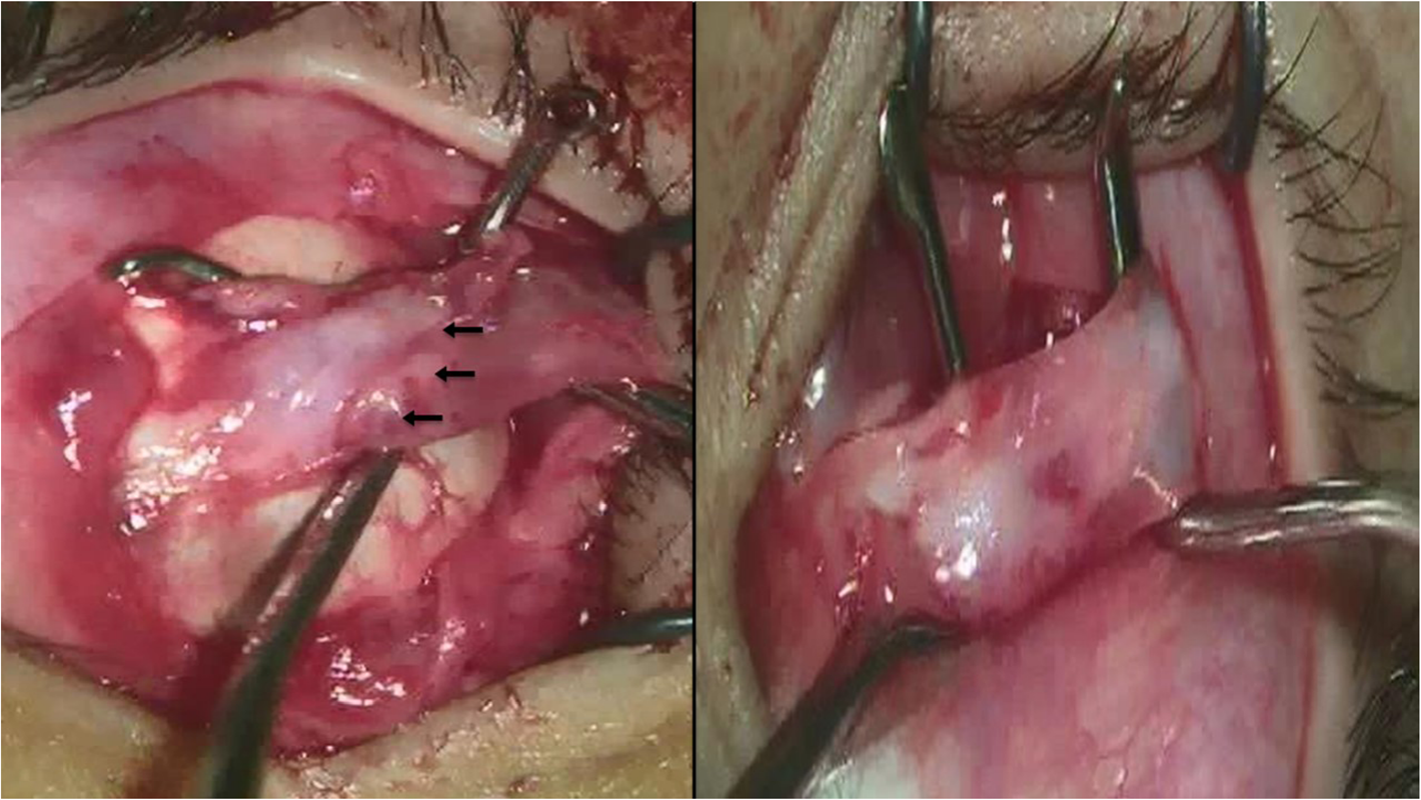
intraoperative appearance of two forms of stretched scar at lateral rectus muscle insertion. (A) a demarcation line could be seen separating the scar tissue form the healthy muscular tissue (black arrows). B, large area of scar tissue which include all visible muscular structures with no demarcation mark. In such case, the tendon-snip test is useful to identify the location of scar-muscular junction

Forced duction test (FDT) was performed immediately before surgery and the results were recorded as positive (mild, moderate, severe) and negative. All surgeries were performed by the senior author (MFF) under general anesthesia using Limbal conjunctival incision with generous posterior release to gain access to the site of the stretched scar. Conjunctival hydrodissection was used in cases with extensive subconjunctival fibrosis and scarring. Site of scleral attachment of the stretched scar pseudotendon was carefully exposed by removing any overlying Tenon’s tissue. Two strabismus hooks were used to isolate the stretched scar-muscle complex from the sclera as posterior as possible using crossed sweeping movement. The stretched scar was identified as glistening whitish bands that sometimes run longitudinally or in other occasions, were grouped to take the whole muscle width. Distinction between normal muscle and stretched scar tissue was done by inspection in cases where scar tissue ran longitudinally and, in such case, the junction was just posterior to where the longitudinal band stopped (Fig. 1a). In cases when broad bands of glistening scar tissue existed, defining the junction by inspection alone was difficult and was aided with the tendon-step test (Fig. 1b). This was performed by sweeping a muscle hook back and forth, under the attachment scar, to identify the step created by the free edge of the thicker, tendinomuscular structure [15].

After identification of the scar-muscle junction, the muscle was sutured just posterior to the junction line in the standard fashion using 5/0 double armed non-absorbable polyester sutures and care was taken to ensure that all scar tissue was anterior to the suture line. If the muscle was previously recessed, the muscle was anteriorly advanced after excision of the scar tissue and was sutured directly to the sclera in the classic crossing-sword fashion. We considered that 1 mm advancement would induce correction equivalent to that of 1 mm resection of the rectus muscle [16, 17], and the amount of advancement was calculated from the standard strabismus surgical tables based on largest angle of deviation measured preoperatively. In case of previous muscle resection, the muscle was sutured directly to the site of original insertion. Conjunctival incisions and occasional buttonholes were closed with interrupted 8/0 vicryl sutures. Postoperative combined steroid-antibiotic drop and ointment was administered postoperatively in slightly higher frequency (5 times per day) and for slightly longer duration (3 to 4 weeks) to minimize the fibrotic healing response. In the early postoperative period, patients were instructed to minimize ocular duction, as possible, by turning their head instead of their eyes to minimize the stretching of any formed scar tissue. Patients were examined postoperatively at first week then at least two times during the first 6 months after surgery. Primary outcome measures were change in the perception of diplopia (if present preoperatively), angle of deviation in the primary position and degree of limited ocular duction. Data were statistically analyzed using the SPSS software program (SPSS for Windows, version12.0 K). Preoperative and postoperative values were compared using paired *t* test with a *P* value less than 0.05 was considered statistically significant.

## Results

During the study period, 27 cases, 17 females and 10 males of secondary strabismus secondary to stretched scar were identified, of them, 16 cases were bilateral. In unilateral cases, the left eye was affected in 7 cases while the right eye was affected in 4 cases. The full data regarding the primary procedures could be obtained in 13 patients only. Out of the study population, 21 cases had consecutive deviations while 6 cases had recurrent deviations. The average age of patients at the time of the primary procedure was 11.4 ± 8.1 years (range; 5–28) and at the time of repair of the stretched scar induced-secondary deviations was 12.1 ± 8.6 years (range; 5.5–29). The average time elapsed between the primary procedures and development of the secondary deviation was 7.7 ± 7.9 months (range; 2–36). Mean spherical equivalent refractive error was − 1.6 ± 2.4 and − 2.2 ± 4.1 diopter in right and left eye respectively. In all patients, the average best corrected visual acuity was 0.7 ± 0.09 and 0.6 ± 0.3 in the right and left eye respectively. Before management of stretched scar, 21 patients were complaining of diplopia which increased in the direction of action of the affected muscle. After surgical correction of the stretched scar, all cases had their diplopia improved. Average follow up period after surgical correction of stretched scar was 10.3 ± 3.1 months (average;6–17). At the final follow up visit, no recurrence of stretched scar- induced strabismus was observed in any patient. All patients were followed up for Patients’ characteristics including angle of deviation, limited ocular ductions both pre- and post-operatively as well as the type and amount of surgical correction are illustrated in Table 1.

**Table 1:** different patients’ categories and postoperative results

Cases	No.	1ry deviation (no.)	1ry procedure (no.)	2ry deviation	Duction	2ry procedure	Final deviation	Final duction
Recurrent deviations								
Recurrent XT	4	42.3 ± 8.6ΔXT (3 cases)	BMR ++ [2] UR&R [1]	53.6 ± 17.2ΔXT	MRU: − 3.8 ± 0.7	scar excision BLR - - 7.3 ± 1.04 m	19.3 ± 11.7ΔXT ***P*** = 0.016	MRU - 0.8 ± 0.6 ***P*** < 0.0001
Recurrent HT	2	HT (N/S)	IR ++	16 ± 2.8ΔHT	IRU: − 2.5 ± 0.7	Scar excision	5 ± 1.4 ΔHT ***P*** = 0.03	IRUA - 0.5 ± 0.7 ***P*** = 0.103
Consecutive deviations								
consecutive ET	15	36.6 ± 11.5ΔXT (7cases)	BLR - - [7]	22.6 ± 5.3ΔET	LRU: −1.7 ± 0.7	Scar excision LR Adv. 4.4 ± 0.5 m	3.5 ± 8.5ΔXT ***P*** < 0.0001	LRU - 0.3 ± 0.5 ***P*** < 0.0001
consecutive XT	6	27.6 ± 6.5ΔET (3 cases)	BMR - - [4] UR&R [2]	52.3 ± 9.5ΔXT	MRU: − 4 ± 1	Scar excision MR Adv. 5.3 ± 0.7 m	13.3 ± 4.2ΔET ***P*** < 0.0001	MRU – 0.5 ± 0.5 ***P*** < 0.0001

*ET* esotropia; *XT* exotropia; *HT* hypertropia; *N/S* not specified; *BMR++* bilateral medial rectus resection; *UR&R* unilateral recession& resection; *BLR* bilateral lateral rectus recession; *BMR* bilateral medial rectus recession; *IR++* inferior rectus resection; *MRU* medial rectus underaction; *LRU* lateral rectus underaction; *IRU* inferior rectus underaction; *Adv* advancement; *MR* medial rectus; *LR* lateral rectus; *IRU* inferior rectus

**Fig. 2:**
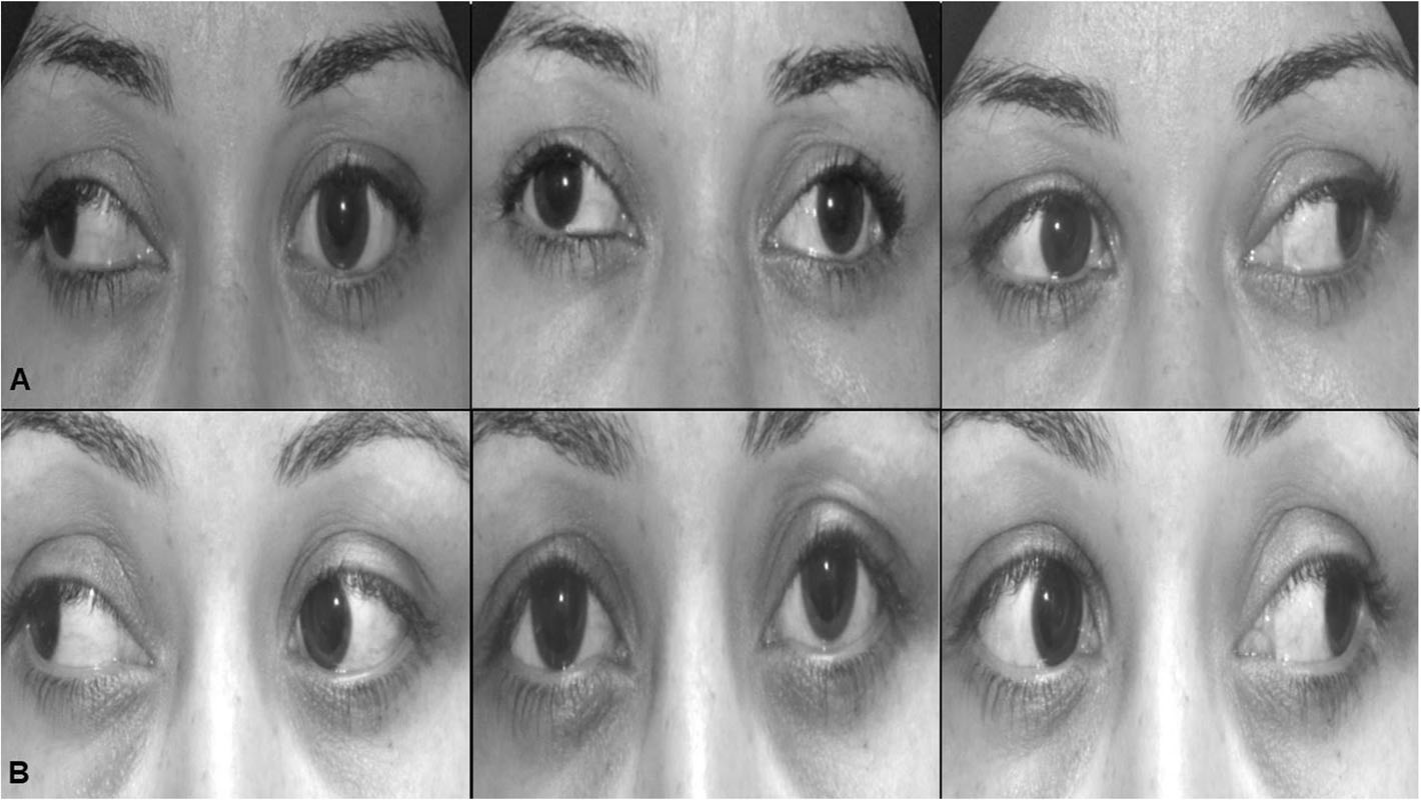
clinical photographs of a patient with stretched scar–induced recurrent exotropia (50 PD) with asymmetric limitation of adduction (right eye: − 4; left eye: − 1), 23 months after previous bilateral medial rectus resection. Excision of the scar tissue, fixation of medial recti to the sclera combined with bilateral lateral rectus recession resulted in improvement of exotropia to 15PD with complete normalization of adduction in both eyes. A: preoperative, B: postoperative. Left column: right gaze, middle column: primary position, right column: left gaze

Cases with stretched scar-induced recurrent deviations (6 cases) fall into two categories: 1) cases with recurrent exotropia (4 cases) which was persisted after previous medial rectus resection (3 cases) and unilateral recession-resection (one case). In those cases, the degree of recurrent exotropia was large enough that mandated additional bilateral LR recession combined with excision of the stretched scar at the medial recti. Postoperatively, recurrent exotropia decreased from 53.6 ± 17.2Δ to 19.3 ± 11.7Δ (*P* = 0.016) while the limited adduction had improved from − 3.8 ± 0.7 to − 0.8 ± 0.6 (*P* < 0.0001) (Fig. 2). 2) Cases with recurrent hypertropia (2 cases) which was persisted after previous inferior rectus resection. Those cases were treated by scar excision at the inferior rectus which resulted in correction of HT from 16 ± 2.8 to 5 ± 1.4PD (*P* = 0.03) and limited depression from − 2.5 ± 0.7 to − 0.5 ± 0.7 (*P* = 0.103).

**Fig. 3:**
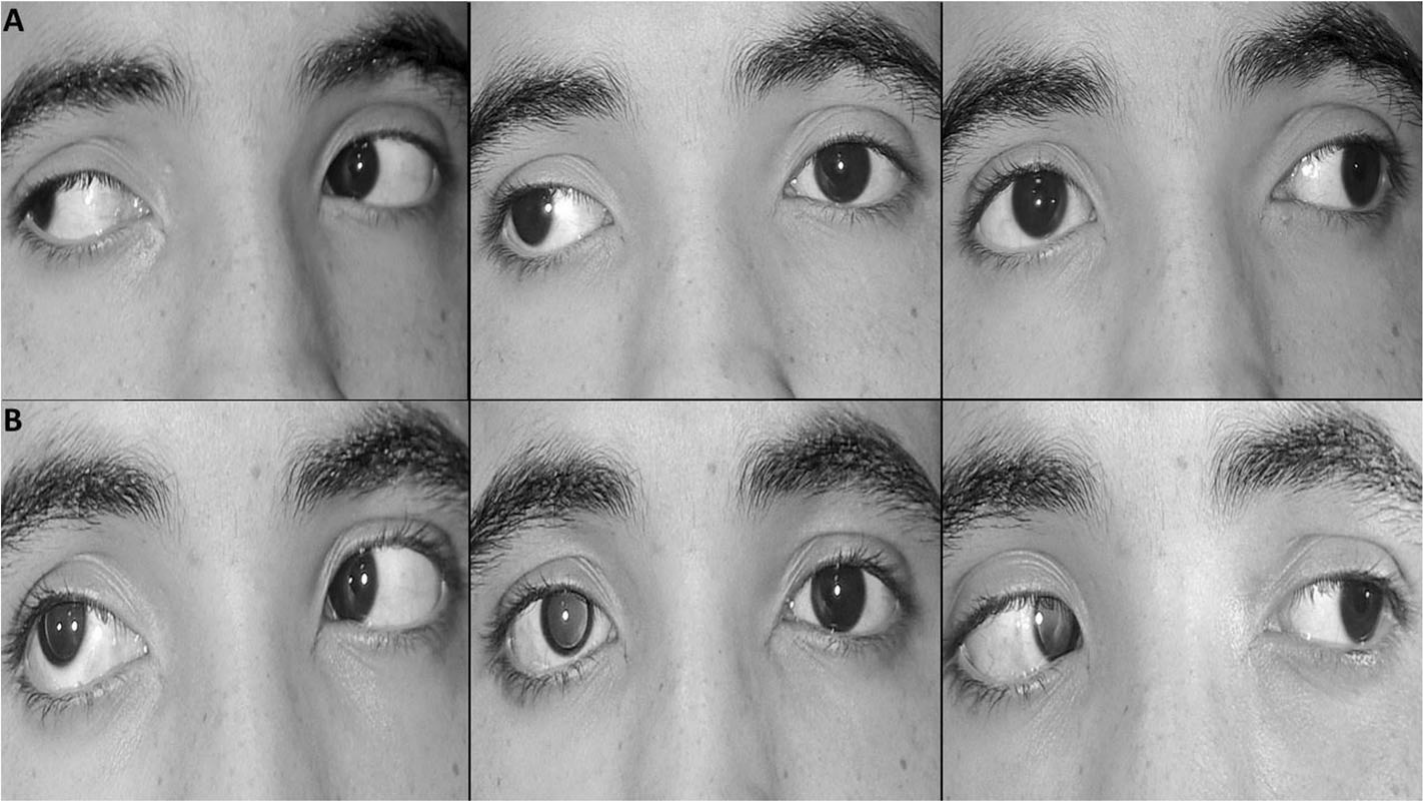
clinical photographs of a patient with a right stretched scar –induced consecutive exotropia 65 PD with severe (− 5) limitation of adduction in his right eye, one year following previous unilateral medial rectus recession and lateral rectus resection. Excision of the scar tissue and 5 mm advancement of the right medial rectus combined with 8 mm recession of right lateral rectus resulted in 12 PD esotropia and 15PD hypertropia with complete normalization of adduction in the right eye. A: preoperative, B: postoperative. Left column: right gaze, middle column: primary position, right column: left gaze. Note, the left pupil was pharmacologically dilated

Cases with stretched scar-induced consecutive deviations (21 cases) also fall into two categories: 1) cases with consecutive esotropia following previous lateral rectus recession (15 cases). In all these cases, excision of the scar tissue combined with advancement of the LR muscles has resulted in significant correction of consecutive esodeviation from 22.6 ± 5.3ΔET to 3.5 ± 8.5ΔXT (*P* < 0.0001) with improvement of limited abduction from − 1.7 ± 0.7 to − 0.3 ± 0.5 (*P* < 0.0001). 2) Cases with consecutive exodeviation (6 cases) following previous medial rectus recession (4 cases) and unilateral recession-resection (two case) (Fig. 3), in whom, XT was improved from 52.3 ± 9.5ΔXT to 13.3 ± 4.2ΔET (*P* < 0.0001) with correction of limited adduction from − 4 ± 1 to − 0.5 ± 0.5 (*P* < 0.0001) after combined excision of the scar tissue and MR advancement.

Perioperative FDT was negative in all but one patient who was presented with consecutive esotropia following a previous unilateral recess-resect procedure for XT. In this patient, whose data was omitted from final data analysis, the FDT was highly positive with severe resistance to abduction which implied severe tightness and restriction of the MR muscle. On exploration, the MR, which was found grossly hypertrophied and tight, was recessed until no tightness was encountered on FDT. No systemic abnormalities were reported that could be linked to the development of stretched scar except for one patient who had an abnormal delayed wound healing following previous abdominal surgery.

## Discussion

Ludwig and Chow introduced the entity of stretched scar as a separate entity in the domain of secondary strabismus [11, 12]. In the stretched scar-induced strabismus, secondary deviations occur months to years after the primary procedure, in contrast to the slipped muscle, in which the secondary strabismus occurs in the immediate postoperative period [13]. This case series shows clinical characteristics and results of management of secondary strabismus caused by stretched scar. Overall, stretched scar-induced secondary strabismus should be suspected in cases with late over or undercorrections associated with limited duction of the previously operated muscle. Management which includes exploration, excision of the scar tissue and reattachment of the muscle to the sclera using non-absorbable sutures is usually effective in restoration of normal muscle function. However, additional surgery of antagonist muscles might be needed in selected cases.

In this case series, the average interval elapsed between the primary procedure and correction of stretched scar induced strabismus was 7.7 months (range; 2–36), a significantly smaller interval when compared with 10, 13 and 29 years that were reported in previous trials [11, 15, 18]. The long-time interval reported in the previous trials could result from the relative non-familiarity of the issue of stretched scar as a cause of residual or consecutive strabismus.

The issue of the stretched scar was addressed in few previous reports. Most of those reports dealt with the stretched scar as a cause of consecutive exotropia following recession of both medial recti for the treatment of esotropia. In one case series, which addressed stretched scar-induce consecutive XT; mean XT was corrected from 33.1PD to 12PD after 4 months of scar excision combined with single medial rectus muscle advancement using absorbable sutures [18]. In the consecutive XT subgroup of the current series (3 cases), mean angle of deviation was improved from 38.4 PD XT to 14.3PD ET after combined excision of the scar tissue and MR advancement. In 2013, Cho and Ryu evaluated their experience in management of consecutive exotropia by MR advancement following medial rectus recession for infantile esotropia. They have reported that slippage of the MR was the cause of consecutive deviation in 17 out of 77 patients [19].

In the past, the underlying pathophysiology responsible for the development of late secondary strabismus associated with weak ocular duction was poorly understood. This had led Cooper [20] to suggest treating cases of secondary strabismus by operating on the fresh muscles instead of the previously operated ones. However, the issue of stretched scar was not clearly understood until the observations of Ludwig and Chow which identified the stretched scar as the culprit for cases of late secondary strabismus associated with poor ocular duction, and also differentiated it from the previously well known “slipped muscle”. Several methods have been used to control excessive fibrosis and scarring which could restrict ocular motility and predispose to postoperative induced deviations, and they include the use of amniotic membrane [21], application of mytomycine-c [22] and injection of triamcinolone [23]. However, their use in regular practice was limited by their complex intraoperative applications, inconsistent results and development of complications [24].

Despite its retrospective nature, small sample size and short follow-up, this trial agrees with the work of Ludwig and Chow in that the stretched scar should be suspected in any case of overcorrection (following previous recessions) and undercorrections (after previous resections) accompanied with any degree of poor muscle duction. Key factors in diagnosis of stretched scar are the late onset and the slowly progressive course of late deviations associated with variable degrees of weakness of ocular duction. Intraoperative forced duction test, which is usually negative in these cases, is used to differentiate stretched scar from tightness and stricture of the antagonist muscle especially if unilateral recess-resect was the primary procedure.

## Conclusions

In the current trial, management of stretched scar yielded satisfactory results. Significant correction of ocular deviation, improvement of diplopia and improvement of limited ocular duction has been observed following excision of the scar tissue with muscle attachment to the sclera using non-absorbable sutures. In general, excision of the scar tissue accompanied by variable degrees of muscle advancement was usually effective in control of consecutive deviations where the involved muscles were previously recessed while in recurrent deviations, where the involved muscles were previously resected, additional surgery on antagonist muscles was sometimes required.

## Data Availability

The datasets used and/or analysed during the current study are available from the corresponding author on reasonable request.
